# Neurogenic thoracic outlet syndrome due to subclavius posticus muscle with dynamic brachial plexus compression: a case report

**DOI:** 10.1186/s13104-015-1317-3

**Published:** 2015-08-14

**Authors:** Julia Muellner, Alain Kaelin-Lang, Oliver Pfeiffer, Marwan Mohamed El-Koussy

**Affiliations:** Department of Neurology, University Hospital Bern, Freiburgstrasse 100, 3010 Bern, Switzerland; Neurocenter of Southern Switzerland, Opsedale Civico, Via Tesserete 46, 6900 Lugano, Switzerland; Department of Neuroradiology, University Hospital Bern, Bern, Switzerland

**Keywords:** Neurogenic thoracic outlet syndrome, Subclavius posticus muscle, Dynamic brachial plexus compression

## Abstract

**Background:**

Neurogenic thoracic outlet syndrome is an underestimated cause of brachial weakness and pain. The subclavius posticus muscle (SPM) is an aberrant muscle originating from the medial aspect of the first rib reaching to superior border of the scapula, which may cause, depending on its activation, dynamic compression of the brachial plexus.

**Case presentation:**

In the present study, we report about a 32-year-old male caucasian patient with weakness in radial deviation of his left hand. An isolated macrodactyly of his left middle finger had been operated twice. Electroneurography showed a carpal-tunnel-syndrome (CTS) on the left side. MRI of the brachial plexus revealed an additional muscle in the costoclavicular space, identified as SPM. To our knowledge, this is the second case report of a neurogenic thoracic outlet syndrome due to SPM, and the first case described with isolated macrodactyly and CTS in the same patient.

**Conclusion:**

If complaints about hand weakness are only reported in cases of distinct hand positions, a dynamic compression of the brachial plexus by SPM may be the cause. A neurogenic thoracic outlet syndrome may facilitate the development of CTS.

## Background

Neurogenic thoracic outlet syndrome (NTOS) is an underestimated cause of brachial weakness, pain and sensory deficits, which is hard to diagnose. The subclavius posticus muscle (SPM) has first been described by Rosenmuller in 1800 [1]. It is an aberrant muscle originating from the medial aspect of the first rib reaching to superior border of the scapula. It may cause dynamic compression of the brachial plexus depending on its activation. Until today, only one other case of neurogenic thoracic outlet syndrome associated to SPM had been described in a patient [2]. However, post-mortal examinations show that this muscle is present in 8.9 % of the individuals [3]. Furthermore, it was already shown, that a neurogenic thoracic outlet syndrome may facilitate the occurrence of carpal tunnel syndrome (CTS) [4]. Here we present a case of an intermittent compression of the brachial plexus due to SPM, associated with CTS and isolated macrodactyly of the middle finger.

## Case presentation

**Fig. 1:**
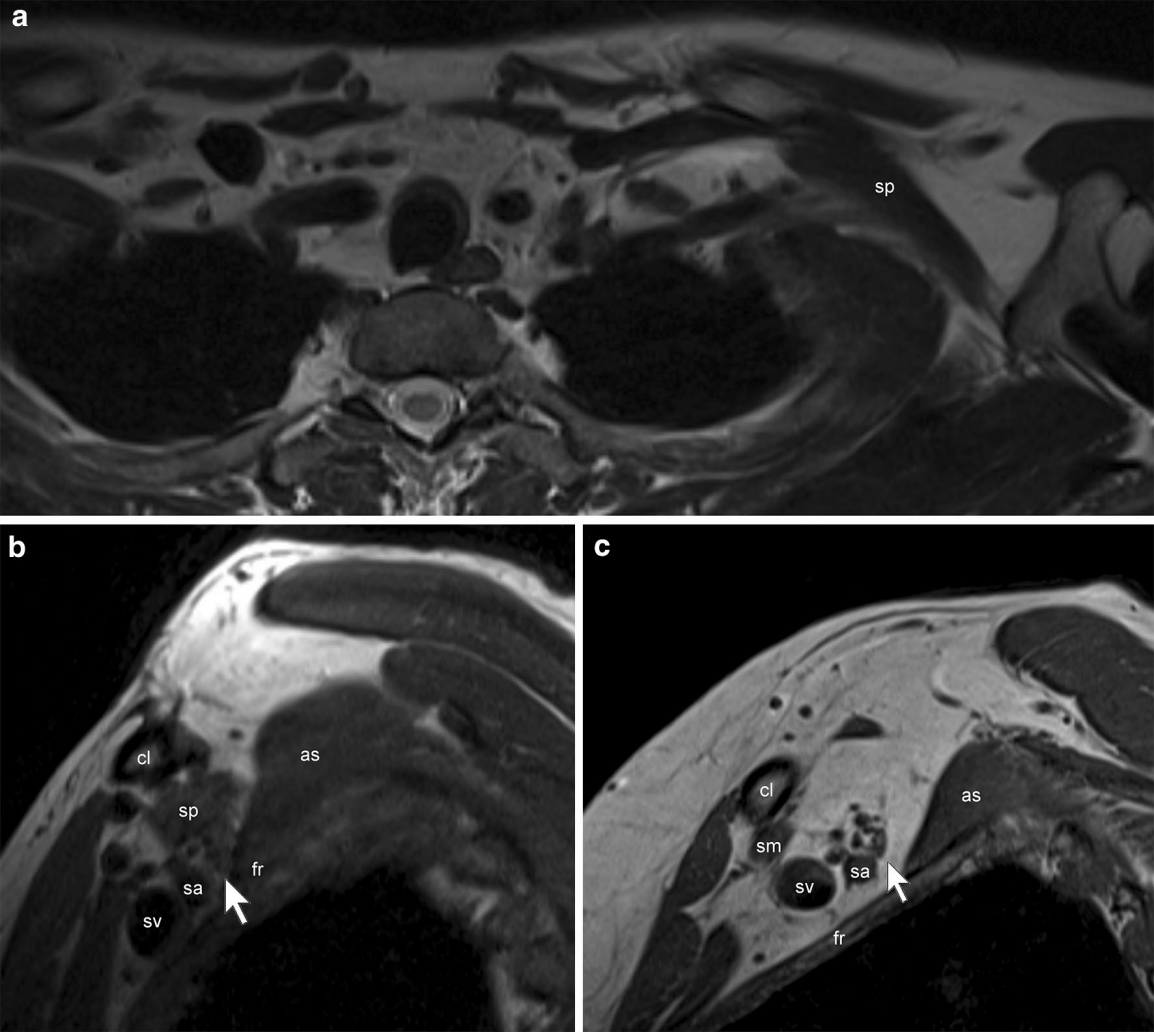
MRI of the brachial plexus at 3T (Magnetom Verio, Siemens, Erlangen, Germany), the arm adducted: **a** axial T2w images showed the accessory subclavius posticus muscle (*sp*) on the left side, attached to the superior border of the scapula and the medial aspect of the first rib (*fr*). No regular subclavius muscle was found. **b** Sagittal T1w images showed the subclavius posticus muscle coursing over the brachial plexus (*white arrow*) and the subclavian artery (*sa*) and vein (*sv*), narrowing the costoclavicular space (*cl* = *clavicle*). There was only a little fat tissue left between this muscle, the neurovascular structures, the first rib and the anterior scalene muscle (*as*). No regular subclavius muscle was found, **c** for comparison a normal appearing costoclavicular space with a regular, thin subclavius muscle (*sm*) of a control subject.

An apparently healthy 32-year-old male patient complained of reduced strength in his left hand. Over the past few months this phenomenon occurred especially and repeatedly when holding a frying pan, but there was no pain or weakness in any of the other muscles. He had two operations on his left middle finger at the ages of 10 and 18 due to a macrodactyly. Neurological examination gave evidence for a reduced sensitivity for touch in his left middle finger. In neutral position, there was no deficit of strength. Electroneurography showed mild CTS, without sign of axonal lesion of the radial nerve. In abduction of the left arm, there was a slight weakness in pronation, radial deviation and elevation of his left hand. Occasional fasciculations were observed in his left brachioradial muscle. Overall, we hypothesized a possible brachial plexus pathology. MRI of the left brachial plexus (Fig. 1) showed an accessory muscle, attached to the medial aspect of the first rib and superior border of the scapula near the base of the coracoid process. The muscle caused a significant narrowing of the costoclavicular space (Fig. 1b). No regular subclavius muscle could been identified on either side. Arm abduction or weight bearing would most likely accentuate the narrowing of the costoclavicular space, with subsequent compression of the brachial plexus.

Overall, NTOS with compression of the brachial plexus which increases with arm elevation and abduction was likely [5]. The muscle having this negative effect was identified as SPM which was first described by Rosenmuller [1] and was recently described in a post-mortem study of a patient suffering from Edward’s syndrome [6]. In this Japanese study [3], 124 corps were examined and SPM was found to be present in 11 patients (8.9 %) and identified as a possible cause of NTOS [7]. The first case report on the presence of SPM in a live patient with NTOS was published in 2010 [2]. NTOS can trigger CTS [4], but CTS may also be associated with macrodactyly [8]. Electroneurographical changes in NTOS may be absent. Therefore, NTOS diagnose depends on clinical examination [9] and imaging [5]. Muscle strength should always be tested in the position in which the patient feels weaknesses or constraints, as SPM is not constantly active [2]. This is in accordance with our patient’s weakness when carrying a frying pan in his left hand, whereas other muscles are at normal strength. According to a telephone interview after 6 months of the MRI examination, he reported that targeted physiotherapy over 3 months resulted in an almost full recovery and loss symptoms.

## Conclusion

This case illustrates that SPM is a rare, but probably underestimated cause of NTOS. Its presence may enable the occurrence of CTS [4] as well.

## Consent

Written informed consent was obtained from the patient for publication of this Case report and any accompanying images. A copy of the written consent is available for review by the Editor of this journal. The consent form signed by the patient is in accordance with the BMC research guidelines.

## Abbreviations

NTOS: neurogenic thoracic outlet syndrome
SPM: subclavius posticus muscle
CTS: carpal tunnel syndrome
